# Online self-administered training for post-traumatic stress disorder treatment providers: design and methods for a randomized, prospective intervention study

**DOI:** 10.1186/1748-5908-7-43

**Published:** 2012-05-14

**Authors:** Josef I Ruzek, Raymond C Rosen, Lisa Marceau, Mary Jo Larson, Donn W Garvert, Lauren Smith, Anne Stoddard

**Affiliations:** 1 National Center for PTSD, VA Palo Alto Health Care System, 795 Willow Road, Menlo Park, CA, 94025, USA; 2 New England Research Institutes, Inc. (NERI), 9 Galen Street, Watertown, MA, 02472, USA; 3 Brandeis University, Heller School for Social Policy and Management, 415 South Street, Waltham, MA, 02454, USA

**Keywords:** Post-traumatic stress disorder (PTSD), Cognitive behavior therapy, Randomized controlled trial, Motivational interviewing, Goal-setting, Behavioral task assignment, Standardized patient, Training, Supervision

## Abstract

This paper presents the rationale and methods for a randomized controlled evaluation of web-based training in motivational interviewing, goal setting, and behavioral task assignment. Web-based training may be a practical and cost-effective way to address the need for large-scale mental health training in evidence-based practice; however, there is a dearth of well-controlled outcome studies of these approaches. For the current trial, 168 mental health providers treating post-traumatic stress disorder (PTSD) were assigned to web-based training plus supervision, web-based training, or training-as-usual (control). A novel standardized patient (SP) assessment was developed and implemented for objective measurement of changes in clinical skills, while on-line self-report measures were used for assessing changes in knowledge, perceived self-efficacy, and practice related to cognitive behavioral therapy (CBT) techniques. Eligible participants were all actively involved in mental health treatment of veterans with PTSD. Study methodology illustrates ways of developing training content, recruiting participants, and assessing knowledge, perceived self-efficacy, and competency-based outcomes, and demonstrates the feasibility of conducting prospective studies of training efficacy or effectiveness in large healthcare systems.

## Background

As a consequence of the wars in Iraq and Afghanistan, increasing numbers of active duty personnel are being exposed to deployment-related traumatic experiences resulting, in some instances, in the development of post-traumatic stress disorder (PTSD) and other mental health problems. Moreover, as a result of Department of Defense (DoD)- and Veterans Health Administration (VHA)-mandated universal screening for PTSD and military sexual trauma (MST), increasing numbers of service men and women and veterans in need of mental health support are being identified. Prevalence estimates for PTSD range from 12% to 20% of returnees from Iraq, and 6% to 11% of returnees from Afghanistan [1]. Dissemination and effective delivery of short-term, efficacious, evidence-based treatments for PTSD is an urgent priority to address this growing public health problem and to prevent the development of costly chronic mental health disorders among returning veterans from the conflicts in Iraq and Afghanistan [2]. Such treatments should also be made available to those experiencing PTSD symptoms as a result of involvement in previous wars.

The internet potentially provides an ideal vehicle for cost-effective training of mental health providers and a potentially effective means to address challenges associated with large-scale face-to-face training initiatives [3]. Once established, online training programs can be delivered on a continuing basis to large numbers of providers with little or no incremental cost. Moreover, internet-based methods can overcome logistical barriers by virtue of their ease of accessibility in the office or home. Despite these established advantages, questions remain about the immediate and long-term effectiveness of internet-based training, particularly in relation to mental health interventions.

Research provides strong support for the effectiveness of mental health interventions based on cognitive-behavioral therapy (CBT) for most psychological problems that bring veterans into care (PTSD, depression, anxiety, and substance abuse), leading to the highest priority for implementation of CBT in the VHA and other healthcare systems [4,5]. In response to the urgent need for large-scale training, several system-wide initiatives have been launched inside the VHA and in other public programs to disseminate CBT interventions for psychological problems [5,6]. Despite widespread acceptance of the clinical relevance and need for these types of interventions, there is no consensus on best practices for accomplishing large-scale dissemination, necessary elements of effective training, or methods of assessing successful outcomes of training [5]. Current research does not sufficiently inform us regarding the effectiveness of web-based training methods either as stand-alone methods, or with supplementation via individual supervision with qualified experts. Post-training supervision (whether face-to-face or remote) is hypothesized to significantly influence behavior of clinician trainees [7], although the empirical support for this assumption is not robust.

The gap between evidence-based interventions and current practice in service delivery has been noted in prevention, chronic care, and many aspects of clinical practice in healthcare in diverse settings and across many disciplines [8-10]. One shortcoming is that many treatment programs fail to adequately identify core assessment and treatment techniques and provide training in those techniques [11,12]. Face-to-face training, while effective in some areas of intervention, is too resource-intensive to provide on the scale required to reach community-based programs [13]. Other lines of research are attempting to improve the transportability of existing CBT interventions by tailoring or optimizing them for delivery in specific systems or for specific audiences, such as with children [14] or veterans [15], by increasing flexibility in implementation [16], and by better integrating cross-cultural awareness into education programs [17]. It is becoming commonplace to incorporate web-based training as one component of dissemination programs for providers in many professions [7,18-21]. The aim of the newest generation of studies is to identify the combinations of training, supervision [22], and support [23] that are necessary and sufficient to maintain good fidelity to evidence-based CBT techniques.

In this paper, we describe the development and testing of an internet-based training program to deliver components of CBT for PTSD, as a stand-alone training and with a group telephone supervision process designed to supplement the training and improve participants’ delivery of CBT techniques.

## Methods

### Study rationale

The overall objective of our study is to conduct a prospective, randomized controlled trial to evaluate the feasibility, implementation, and effectiveness of internet-based training in CBT techniques, including a standardized telephone-delivered supervision method. Secondary aims of the study are: to develop a module-based online training program in CBT techniques that is sufficiently engaging to encourage maximal participation; to recruit a large, diverse sample of mental health providers for participation in training research under ‘real world’ conditions; to design and administer web-based surveys to assess knowledge acquisition and changes in perceived self-efficacy; to design, implement and evaluate a structured method for telephone-based case supervision; and to develop and implement a standardized patient behavioral observation assessment methodology for evaluating skills performance during simulated treatment delivery.

### Rationale for selection of core training modules

The CBT techniques were divided into three training modules: motivational interviewing; goal-setting; and behavioral task assignment. These modules were selected for multiple reasons. First, all three modules focus on the essential need to prompt behavior change in those seeking treatment services. Second, they represent behavior change skills that can be trained with relatively brief investment of time and effort for potentially large yield in clinical performance and outcomes. These core techniques and practices are typically embedded in multi-session CBT package interventions, which made conversion to web-based training format readily feasible for the current study. Third, the modules we selected focus on generic, multi-purpose CBT techniques, which can be broadly applied across a wide range of behavior change targets. Finally, these techniques can be integrated by diverse providers into ongoing therapy sessions flexibly and immediately. The practices to be disseminated were also considered to ‘fit’ well within existing structures of PTSD care within VHA (*e.g.*, time-limited approaches), an important consideration in dissemination, and to reflect expressed training interests among PTSD treatment providers who participated in focus groups. Generally, the three techniques have not been studied as stand-alone interventions for PTSD, and they are not designed to represent comprehensive PTSD interventions. Motivational interviewing has been used successfully to increase motivation to change a range of behaviors that co-occur with PTSD, whereas goal-setting and behavioral task assignment are components of existing evidence-based PTSD protocols (*e.g.*, prolonged exposure and cognitive processing therapy), as well as interventions for other trauma-associated problems.

Experience within VHA PTSD treatment programs indicates that many veterans fail to engage in treatment, drop out after attending one or two sessions, or are reluctant to fully participate (*e.g.*, attend group sessions). Cognitive-behavioral motivational interviewing using the widely tested methods of Miller and Rollnick [24] was therefore selected as one empirically-supported technique that providers could employ to help motivate veterans to participate in the treatment process. This technique also helps providers respond to patient ambivalence about changing key PTSD-related behaviors, such as substance abuse, maladaptive weapon storage, and violent behavior, all prevalent among returning service members with PTSD [25].

Goal-setting was selected as a core technique because delivery of CBT interventions is predicated on a process of mutual patient-provider negotiation of treatment goals. Nevertheless, in routine clinical care, this mutual and systematic negotiation of short-term treatment goals often does not occur. Instead, patients are offered a set of standard treatment options (*e.g.*, participating in a PTSD education class, receiving a stress management intervention). It is likely that creation of individualized goals will increase patient engagement by maximizing the personal relevance of treatment. Such a client-centered approach also increases the likelihood that the patient will attempt behavior change and attend treatment sessions. For the mental health provider, such goal-setting helps to ensure ‘customer-focused’ treatment.

A core technique in CBT interventions is behavioral task assignment that facilitates transfer of training out of the office into the real world environment of the patient. Patients who work to change their behavior outside of the counseling session can be expected to accomplish more significant change. They can achieve concrete benefits and increase their perceived self-efficacy in coping with their life challenges. As patients complete self-monitoring assignments via handouts and forms, they can more systematically learn about themselves and provide the clinician with clearer accounts of between-session behaviors. The provider reinforces successful task performances and works with patients in a variety of ways to reduce non-adherence. Thus, this CBT technique was selected for web instruction because it is versatile and can increase the impact of routine clinical interactions, and because many providers are not currently using behavioral task assignments.

### Format of the training modules

The CBT training modules were designed to be brief, with each requiring approximately 80 min online to complete. Components of the training modules include:

1. Learning objectives that outline the expectations for each module;

2. Interactive components that illustrate key points and demonstrate real-world application of the techniques (*e.g.*, via audio-streamed modeling);

3. Downloadable handouts that summarize content for providers;

4. Downloadable patient education materials that accompany the provider content; and

5. A final knowledge quiz enabling participants to qualify to receive continuing education (CE) credits.

Case examples were also carefully developed to illustrate practical aspects of implementation and challenges commonly faced by providers. In essence, these cases provide authentic examples of how patients actually present.

### Content development

Development of the training modules required several steps to ensure that the content was created in conjunction with the latest behavioral evidence, that the information delivered was balanced, and that the participants were provided with necessary information to be considered trained in these core aspects of CBT. Experts in the techniques (motivational interviewing, goal-setting, and behavioral task assignment) were consulted to provide content oversight on the core modules. Once the module drafts were complete they were reviewed by study staff and by additional experts in CBT who had not been involved in the development of the modules. This important step ensured an independent review of the content. Once modules were reviewed and approved, a final script was created and reviewed a final time to ensure that the integrity of the initial content document was maintained after adding interactive elements and formatting text for the web.

**Figure 1:**
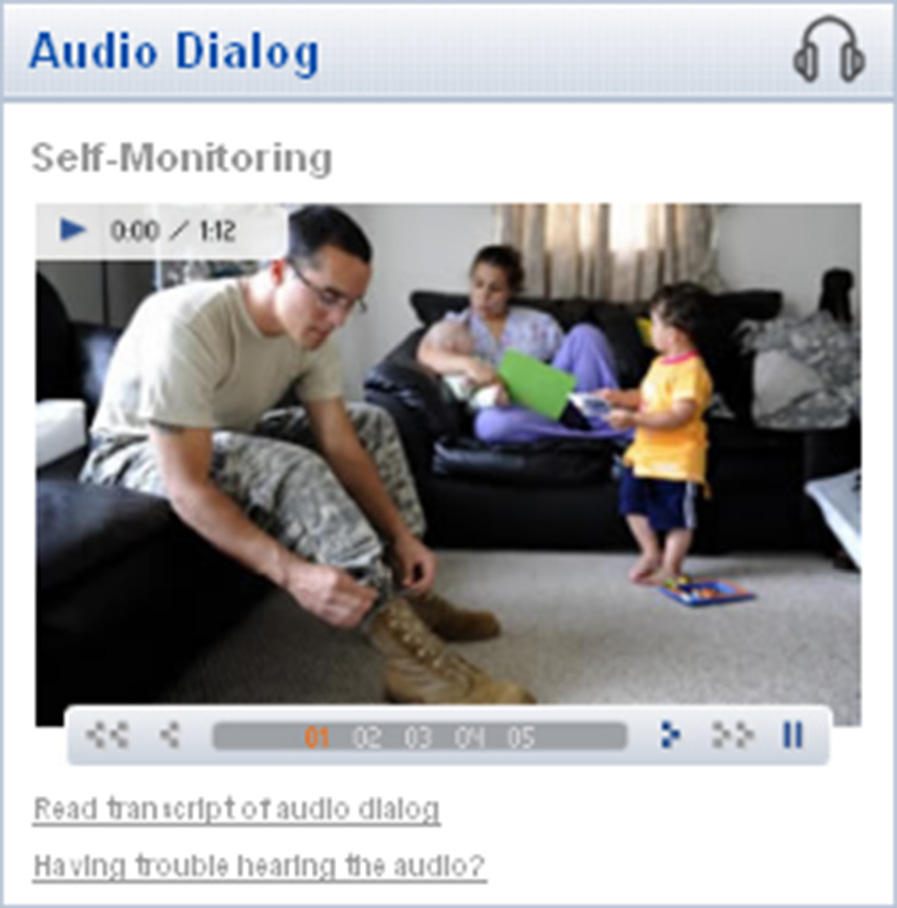
Example of audio dialog with photo slideshow.

**Figure 2:**
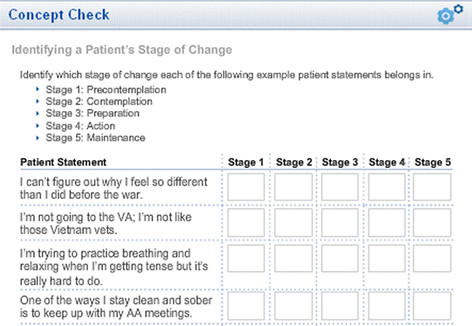
Example interactive exercise.

Each module included interactive audio elements in which a case interaction was presented to demonstrate appropriate and inappropriate approaches to technique delivery (See Figure 1). Course modules also included interactive exercises (Figure 2) that reinforced key concepts listed in the learning objectives and presented information in an easily accessible PDF format for worksheets and other training-related documents.

While content development was being conducted by the authors, the website graphic design was created, reviewed, and approved, creating a distinctive ‘look and feel.’ Formatting of interactive elements (audio, PDF styles, and animation) was conducted and all materials were developed according to guidelines to make electronic information accessible to people with disabilities (508 compliance) available at the time of the study. Several quality assurance steps were conducted to ensure that the program functioned properly. Testing was conducted on each completed module to ensure that all content flowed properly, and the entire site was subject to a formal quality review prior to launch.

### Telephone supervision process

Supervision was aimed primarily at assisting providers in learning and implementing new techniques acquired from the web-based CBT training. During the supervision sessions, providers were guided in implementing specific techniques in a routine manner in their VHA treatment setting and in fully grasping the theoretical and practical aspects of these new techniques. Studies of the supervision process have suggested that cognitive-behavioral supervision in which supervisors monitor the clinician trainee, model competent technique delivery, give specific instructions, set goals, and provide feedback on performance are associated with benefits to clinician trainees [26]. Supervisors were encouraged to apply the same core CBT techniques of motivational interviewing, goal-setting, and behavioral task assignment in their own work with clinician trainees during supervision phone calls. That is, supervisors were asked to model the techniques during their interactions with clinician trainees.

To translate to larger-scale training efforts, a system of supervision must be feasible to deliver, brief enough to encourage clinician trainee participation and completion, and sufficiently comprehensive to ensure learning of new techniques. Given the prohibitive costs of developing local supervision capability for all VHA locations to ensure access to face-to-face supervision, if supervision is to be delivered at scale, it will likely need to be offered at a distance via telephone or videoconferencing. The model of telephone supervision described here was intended to represent a ‘middle-ground’ approach that might make a meaningful impact on participant skills and implementation, while limiting costs and staffing resource demands of supervision. An intensity of five to six weekly supervision sessions, each lasting a maximum of 60 min, was selected on the assumption that a less intensive model would have limited impact, while requiring more sessions might place excessive demands on participant time. Because VHA providers most often schedule for 50-minute treatment sessions, these time frames fit well with scheduling for participants.

Supervisors were selected from National Center for PTSD Dissemination and Training Division staff. They had doctorates in clinical psychology and were familiar with cognitive-behavioral psychological theory and treatment methods. Several had extensive experience in conducting training workshops and were experts in specific CBT interventions (*e.g.*, prolonged exposure, acceptance and commitment therapy).

To ensure a structured supervision process, a manual for providing clinical supervision via telephone was developed. Training for supervisors included: completion of the web modules; review of important theoretical and empirical background information about CBT; completion of a one-day training including a refresher on the three core CBT techniques; training in supervision methods via lectures and video illustrations; and practice in giving feedback on therapy sessions. Supervisors also participated in bi-monthly teleconference calls to obtain regular feedback on their supervision of newly trained CBT therapists.

Six primary supervisors and two back-up supervisors were available to conduct calls. Supervision was scheduled using a cohort design, in which each supervisor was expected to complete six small-group calls over six consecutive weeks with the same cohort of clinician trainees. Each cohort comprised one to four clinician trainees and new groups were started every week. Order of supervisor participation was chosen in advance, and each newly-formed cohort was assigned to the next-in-line supervisor. Back-up ‘on-call’ supervisors were asked to cover for primary supervisors unable to make their regularly scheduled calls due to illness or other unforeseen problems.

Immediately after completing the web training, participants assigned to the web-based training plus supervision group received an email description of the format and procedures of supervision, self-monitoring homework forms, and confirmation of their first consultation session.

Clinician trainees were asked to self-monitor their application of the core techniques via systematic use of a CBT session checklist created for the project. This self-monitoring activity was expected to help focus clinician trainees and supervisor attention on core aspects of the techniques. A CBT session checklist was the primary means for assessing adherence to elements of the techniques during the study, and both clinician trainees and supervisors were expected to have a copy of the form in front of them at all times during supervision calls. Clinician trainees were expected to submit these checklists to their supervisors prior to each supervision call via fax or email. Supervisors were expected to review clinician trainees’ forms before each supervision call and use the information to set a focused agenda for the session. As an additional teaching aid, clinician trainees were asked to complete some of the same homework forms that are typically assigned to patients as a way to track their own clinical behaviors/skills. A ‘Monitoring Your Goals’ form was used for general tracking of clinical goals over the week between supervision calls and a task assignment log was used to track specific behaviors (*e.g.*, the clinician trainee’s use of the motivational interviewing ‘personal ruler’ technique). To meet the criterion for completion of the supervision process, participants had to complete three or more of the possible six supervision sessions.

### Recruitment and randomization

PTSD treatment providers working in active treatment settings were recruited to test the continuing education course modules. To be eligible for study participation, a provider had to be employed full-time as a VHA provider in a mental health clinic, and be treating patients with PTSD at the time of recruitment. VHA protocol prohibits compensating staff members for research participation; however, in addition to the opportunity to learn the new techniques presented in the training, all participants were offered the opportunity to earn four professional continuing education (CE) credits. Those assigned to the training-as-usual (control) group were able to complete the training and receive continuing education credits after their participation in the study was completed.

**Figure 3:**
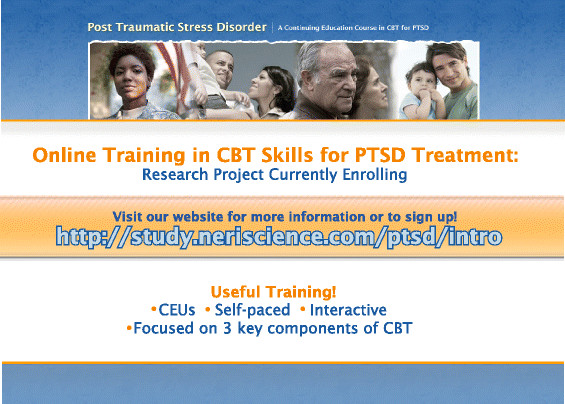
Recruitment postcard.

Because of restrictions on participant compensation, there was an initial concern that, for busy providers, recruitment might be challenging. Thus, careful attention was paid to recruitment activities and the opportunity to participate in the research project was announced in several forums. Study fliers (Figure 3) were produced and distributed at two VHA mental health conferences and at several training events (*e.g.*, National Center for PTSD Clinical Training Program). Four email lists of mental health providers across the country were compiled and sorted randomly to avoid any potential biases. To minimize selection bias, during each week of the recruitment phase, approximately 50 providers per week were contacted from the four lists equally, and invited to participate. Providers responding to the announcements accessed the study website, where they were introduced to the study process, completed brief online questions that screened for eligibility, and if eligible, completed an online consent process.

Once consented, participants were randomly assigned to one of three study conditions: web-based training plus supervision, web-based training, and training-as-usual (control). Participants who completed the baseline assessment, including the simulated patient interview, were stratified by self-reported previous CBT experience and randomized to one of the three training groups. We used permuted blocks of nine participants within each stratum. The control group clinician participants were free to participate in any training activities they would otherwise receive, including local continuing education activities, conference participation, or other formal training programs taking place in the VA. Participants were stratified on the basis of self-rated degree of expertise in cognitive-behavioral therapy (low = none or beginner; high = intermediate, advanced, or expert) and were randomized within strata to one of the three training groups (See Table 1)**.**

**Table 1:** Stratified random group assignment

	**Self-rated CBT expertise**	
**Random Group Assignment**	Low	High
Training-as-usual	19	37
Web-based training only	21	36
Web-based training +expert supervision	19	36

In this study, a web-based screening instrument was developed and made available through a recruitment registration website announced on flyers and in recruitment emails. Participants accessed the online screener that determined eligibility for the study. Individuals were excluded if they: were not a full-time VHA or Vet Center employee; were not a clinical psychologist, social worker, psychiatrist, readjustment counselor, mental health counselor, substance abuse counselor, nurse, psychology trainee, or a trainee classified as ‘other’; were not currently counseling/treating patients with PTSD as part of their VHA or vet center clinical work; or had previously completed VHA-offered training in cognitive-behavioral therapy for depression (whose content domains overlapped significantly with web module content).

**Figure 4:**
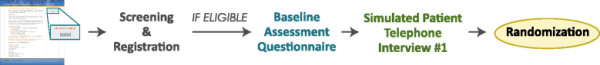
Screening and enrollment process.

Those eligible were directed to an online informed consent process. If consent was completed (requiring two online signatures), the baseline assessment instrument was made available. Those not eligible were thanked and informed that they were not eligible for the study. A web-based baseline assessment was administered to determine baseline knowledge and perceived self-efficacy. In addition, participants had to schedule and complete a standardized patient interview (SPI, Time 1, discussed below). Both the baseline assessment and SPI were required prior to randomization and access to the web training. Randomization to one of the three groups occurred once the participant was identified as having completed the SPI. This process is illustrated in Figure 4.

**Figure 5:**
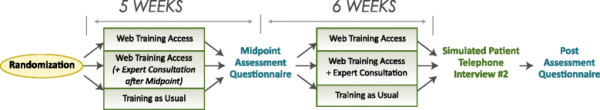
Study design.

If the participant was randomized to either of the web-based training groups, a user name and password was automatically generated and sent to the participant’s email. Participants in the two web-based training groups had a five-week window to complete the web training modules at their own pace and in any order before being prompted to complete the midpoint assessment. When the midpoint assessment was due, access to the web training was suspended until the assessment was completed. After completion, the web training was again made available. The midpoint assessment included an evaluation of the training and assessment of knowledge (which also served as the CE quiz). This midpoint assessment also served as a trigger for the web-based training plus supervision group to begin supervision calls. Participants randomized to web-based training (without supervision) were allowed continued access to the training materials but had no other additional contact until the end of the six-week supervision period. Participants randomized to web-based training plus supervision began scheduling regular supervision calls over a six-week period. At the completion of the six-week supervision period, participants in all three groups were contacted by email to begin scheduling the second SPI (Time 2). Immediately following completion of the second SPI all participants were sent an email directing them to the web-based post-training assessment. Completion of the post-training assessment concluded study participation. At this point, training-as-usual (control) group participants were provided access to the training and were offered the opportunity to complete the CE questionnaire for credits. This overall process is illustrated in Figure 5. Ethical review and approval for the study was provided by the Stanford University and NERI Institutional Review Boards and the VA Palo Alto Health Care System Research and Development committee.

### Assessment methods

#### Evaluation strategy and procedures

**Figure 6:**
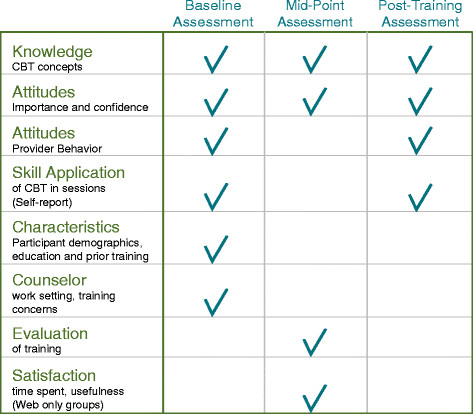
Web-based research measures.

Assessment of effectiveness of online training is intrinsically challenging. Assessment methods in large healthcare systems must be easily implemented, relatively brief in duration, and scalable at modest cost to reach large numbers of geographically-distributed providers. In the current study, online self-report questionnaire methodology provided a relatively simple way of assessing results of training for a large number of mental health providers. Three core domains of outcome were measured: knowledge assessed through multiple-choice tests; self-reports of perceived self-efficacy in technique application; and to assess implementation of CBT techniques, self-reported frequency of application with PTSD patients. The content of web-based data collection and the assessment time points are shown in Figure 6.

While self-report instruments can assess knowledge, perceived self-efficacy, and participant-perceived degree of use of CBT techniques, they are limited in their ability to examine actual skills development. The latter outcome is, of course, especially important in evaluating the success of training. To assess actual mastery of therapy skills, our primary outcome measure was based on simulated treatment-delivery interviews with standardized patients. Standardized patients have been used widely in medical settings to teach and measure skills of assessment and diagnosis [27,28]. This methodology has rarely been used to examine intervention skills. However, a small but growing literature exists for the use of standardized patients in mental health training, particularly to evaluate clinical interviewing for substance abuse [29,30]. We conducted the standardized patient interview sessions over the phone, to facilitate assessment of mental health providers located across the country. This combination of online and telephone assessment methodologies was deemed practical to deliver and complementary in terms of information yield.

#### Standardized patient (SP) interview methodology

Measurement of participant performance in simulated sessions with the standardized patient was used to assess actual skills acquisition. The goal was to create an assessment protocol that ensured standardization of stimulus conditions while permitting actors in the standardized patient role to respond with considerable flexibility depending upon which of a wide range of provider behaviors occurred. The standardized patient interviews were conducted at baseline and follow-up for all participants.

Participants received email orientation materials 72 h prior to their interview. This orientation included a brief description of procedures for the standardized patient interview, description of the clinical background of the patient, information about the time and date of the phone interview, and the phone number at which they would be contacted. 24 h prior to the scheduled interview, the actor was instructed to call the participant and remind him or her to read the materials and confirm when the interview would take place. If the participant did not answer this call, the actor was instructed to leave a voicemail or a message with the receptionist if voicemail was not available.

Simulated interviews were conducted during 50-minute telephone calls. The 50-minute sessions were divided into a five-minute introduction/orientation followed by three 15-minute segments (one for each intervention). We defined a segment as a portion of the standardized patient interview that could be rated according to study criteria.

**Figure 7:**
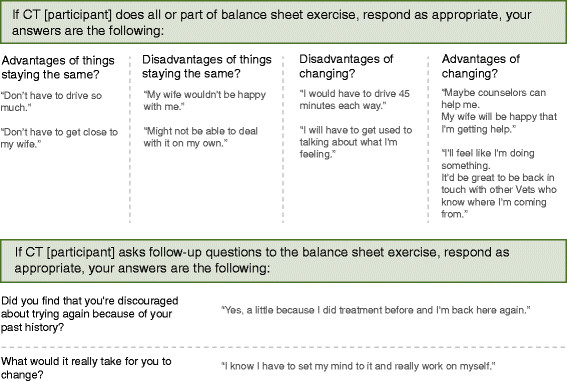
Example conditional responses from standardized script.

To ensure a uniform set of stimulus conditions, a scripted approach to the simulated interviews was developed. Upon initiation of the interview, scripted actor statements were used to orient the participant to the process, and ensure readiness to participate in the interview (*e.g.*, to ensure that the participant had read the patient background material, and was located in a quiet environment with relative protection from interruption). Standardized scripts were also used to open the three segments. In this way, it was possible to convey standard presentations across conditions and actors and provide participants with standardized opportunities to demonstrate appropriate techniques. Because of the potential for variation in participant behavior, conditional responses (Figure 7) were developed to guide actor response after the opening statements. The rules were kept relatively simple in order to avoid overloading the actor with performance demands.

Effective actor selection and training were critical to ensure authenticity of the interaction. To ensure that actors were familiar with the clinical presentation of PTSD in veteran patients, clinical psychology graduate students were interviewed for potential participation. Those selected had typically worked in a VHA practicum setting and performed well on a practice role play that was part of the selection process.

Actors were given a manual describing the interview procedures. The manual included background information about the patient they would portray, an overview of the procedures for the interview, a telephone script for the one-day reminder phone call about the interview, the interview script (including general instructions and scripted statements), conditional responses, and technical instructions for audio recording the interview. Background information about the patient was intended to provide the actors with enough information to complete their interactions with participants in a manner consistent with natural therapeutic conversation and allowing flexible responses to questions. Background description included details of personal history, medical history, symptom display, reasons for seeking help, personality (*i.e.*, response style), affective presentation, and general disposition; these areas were covered in anticipation of possible questions and statements produced by participants. Actor training sessions were held in close time proximity to the conduct of the interviews. Each actor completed a two-day intensive in-person training workshop where they were given an overview of the study protocol, watched video footage of veterans with PTSD, participated in a question-and-answer session with a veteran who had recently graduated from a local inpatient PTSD program, reviewed the protocol for each segment of the interview, and then participated in numerous role-plays. Actor trainees were given significant opportunity to practice each of the techniques in the simulated interview and receive feedback on their performances. They worked as a team during practice sessions to observe one another’s performances and decide if any alterations needed to be made to the standardized patient protocol.

After it was judged that the standardized patient protocol and actor performances were adequate, ‘dress rehearsals’ were conducted in which the actors called study personnel who played the role of study participants. Actors were required to demonstrate their fidelity to the interview structure as well as ability to successfully record the session and deliver the recording to the study manager.

**Figure 8:**
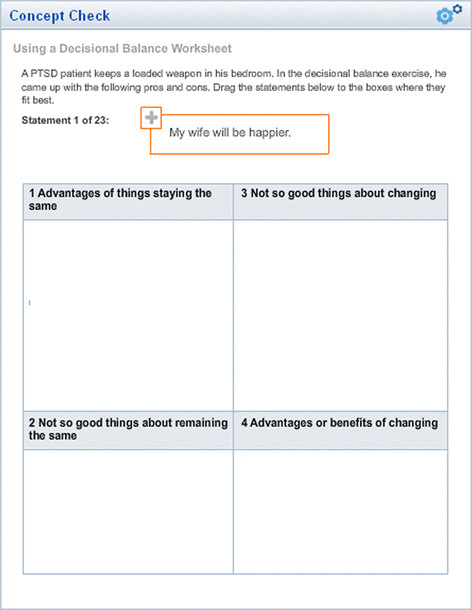
Decisional balance interactive exercise.

The script developed for the standardized patient interview included instructions for the conduct of the interview, and an outline of the clinical details and instructions for each of the three interview techniques (motivational interviewing, goal-setting, behavioral task assignment). Furthermore, opening statements were scripted to begin each of the segments. In the case of motivational interviewing, two additional scripted statements were included to provide opportunities for participants to respond to deliberate change and counter-change statements. Conditional responses were designed to facilitate anticipated techniques taught in each of the respective modules. To maintain consistency, the actors were trained to identify and respond to some of the specific techniques with scripted responses. For example, in the motivational interviewing module, participants were taught to use a decisional balance sheet exercise (Figure 8), and actors were trained on the conditional responses illustrated in Figure 7**.** Actors were blind to the condition to which the participant was assigned.

#### Rating of simulated interview performances

Raters were graduate student research staff not delivering the intervention, all of whom were masked to study condition and time point of assessment. Five practice transcrips were rated independently and discussed as a group to identify points of convergence and divergence. Decisions about rating disagreements were reached and clarifications were added to the rating guide. Once rating began, 20% of all transcripts were randomly selected to be re-rated by a second rater. Raters were masked as to which transcripts were being re-rated.

For each of the CBT techniques, raters scored transcripts based on the degree to which specific technique-related participant behaviors were observed. Each behavior was rated as ‘0’ (Does Not Employ), ‘1’ (Partially Employs), or ‘2’ (Consistently Employs). Behavioral criteria for the three rating levels guided raters. For motivational interviewing, goal-setting, and behavioral task assignment, 17, 8, and 15 behaviors, respectively, were rated. Examples of techniques for behavioral task assignment were ‘frame as an experiment’ and ‘generate potential tasks.’ For each of the three techniques, raters also scored ‘overall degree of collaboration’ (0 to 4) and ‘overall effectiveness’ with the techniques (0 to 4).

#### Statistical analysis plans

##### Primary analyses

The primary outcome of the study intervention is the performance of participants in demonstrating CBT techniques during the SPI assessment at post-training. Summary scores for each of the CBT techniques will be constructed from the ratings of the SPIs and the properties of the scores evaluated. The analyses to be performed will compare the performance of participants in the training-as-usual (control) and web-based training groups using the analysis of covariance (ANCOVA) comparing the average post-test scores among the three training groups, adjusting for baseline scores [31]. We hypothesize that participants in the web-based training groups will exhibit greater improvement in skills performance than those in the training-as-usual (control) group. We also hypothesize that web-based training plus supervision will be associated with greater skills improvement than web-based training alone. If the null hypothesis of no difference among the three groups is rejected, we will compute pre-planned comparisons of the scores between the individual groups adjusting for multiple comparisons. All participants will be analyzed in the training group to which they were randomly assigned, regardless of their compliance with the assigned training method and the assessments. Participants who do not complete the post-training SPI will be considered to have the same value at post-test as at baseline. To assess sensitivity of our results to potential response bias due to more ‘successful’ trainees completing the follow-up assessment, we assumed that non-completers made no change from baseline (last observation carried forward). This is a commonly used method of imputation when doing this type of sensitivity analysis.

Analyses focusing on the intent-to-train sample will also be repeated for the sample of participants who completed ‘therapeutic doses’ of the training interventions. To meet the criterion for completion of the web portion of the training, a participant will have to complete 75% of each of the three modules. To complete the web-based training plus supervision process, a participant will have to complete 75% of each of the online modules and attend three or more of the supervision sessions.

##### Secondary analyses

We also hypothesize that providers in the web-based training groups will show significantly greater improvement in CBT-related knowledge, perceived self-efficacy, and self-reported implementation of CBT techniques than those in the training-as-usual (control) group. The baseline and post-test assessments consist of multiple choice knowledge items, perceived self-efficacy, and practice behavior variables. As with the SPI scores, summary scores will be calculated from the individual questions. Treatment groups will be compared using ANCOVA on the post-test scores controlling for the baseline scores with follow-up comparisons as described above. Moderator analyses are not planned given the use of a randomized trial for testing the primary and secondary hypotheses of the study.

##### Power analysis

Power calculations were computed based on the estimated percentage of participants who would successfully implement the techniques as assessed by the SPI. These calculations indicated that 40 participants per group (total n = 120) would provide 79% power to detect a rate of successful implementation of 48% in the web-based training (without supervision) group compared to 15% in the training-as-usual (control) group, and 91% power to detect a rate of 54% in the web-based training plus supervision group compared to 15% in the training-as-usual (control) group, both at the 2.5% significance level.

## Results and discussion

**Figure 9:**
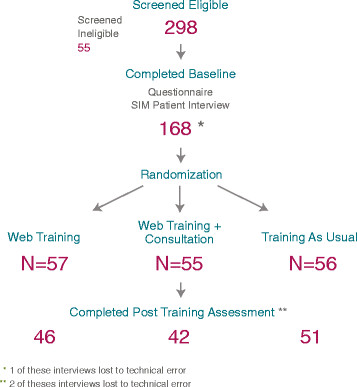
Flowchart of participant recruitment and study completion.

In this paper, we present evidence for the effectiveness of our study recruitment procedures. As shown in the flowchart of recruitment and study completion in Figure 9, we exceeded our recruitment goals. The study protocol called for recruitment of an approximate target of 135 mental health providers; in fact we were able to randomize 168 participants for the study, ahead of the planned recruitment pace. In keeping with the overall composition of VHA mental health providers, our sample was predominantly white (79%), female (70%), and had post-graduate training in psychology, social work, nursing or psychiatry. The average age was 49, and more than 90% had post-graduate qualifications (Master’s or Doctoral level).

Recruitment was scheduled to take place over the course of 12 months; however, due to the rapid success of the recruitment process, full recruitment was achieved in nine months, with an average recruitment rate of 19 participants per month. Recruitment began to exceed targeted numbers in month two of the project, and continued to exceed targets during the successive months. This is a key indicator of both perceived need for web-based CBT training and feasibility of recruitment. Effects of the training interventions on the range of outcome variables will be the focus of presentation in our forthcoming papers.

## Discussion

There are few randomized, controlled trials of training interventions for best practices in mental health, and still fewer outcome studies of web-based training methods. While web-based interventions have been shown to be effective in changing multiple health behaviors in diverse patient populations [32], few studies to date have examined their use in the context of training for trauma intervention among practitioners. We present here the design, rationale, and methods for the first randomized, controlled trial of web-based training for mental health providers providing clinical care to veterans with PTSD. The central question to be addressed is whether web-based training, with or without individual supervision, may provide an effective means to train increasing numbers of mental health providers in relevant, evidence-based clinical techniques. The lack of controlled outcome studies of evidence-based training in mental health practice is of special concern, given the urgent need for increased clinical services in this area [3]. Web-based training interventions, in particular, offer substantial potential for cost-effective application to a broad, multi-disciplinary audience of mental health providers.

The training component of the study is innovative in being entirely web- and telephone-based, and therefore easily accessed and completed by participants. Importantly, it delivers instruction in core techniques (*e.g.*, motivational interviewing) that have traditionally been acquired via one-to-one or small-group in-person training, methods that effectively limit ability to train mental health providers in adequate numbers to address current clinical needs. Moreover, this study addresses the important comparative effectiveness analysis initiative put forth by the U.S. Federal Government [33], which promotes the rigorous evaluation of different interventions to ensure that patients receive the most effective and cost efficient medical care. Recognizing that effective diffusion of robust evidence-based interventions into everyday practice is as essential as development of successful interventions themselves [34], the approach was developed to allow maximum transportability and accessibility for research or training purposes. To ensure evaluation of a supervision model that is maximally transportable, we chose not to include audio or video recording of participant clinical sessions for review by supervisors. Although such review is likely to improve the quality of supervision, it requires time and effort on the part of providers and supervisors that raises the burden of the training process and may be difficult to implement widely on a routine basis. The trial itself combines elements of effectiveness (*e.g.*, participants were providers operating in routine client care settings, supervision procedures were designed to be easy to implement and limited in terms of their time and effort burden) and efficacy research (*e.g.*, careful measurement of skills improvement, random assignment to training conditions). We plan to report analyses of both intent-to-train and completer analyses, with the latter providing more information about the efficacy of the training interventions.

Training addressed three core techniques for achieving behavior change: motivational interviewing, goal-setting, and behavioral task assignment. The three techniques were selected as applying broadly across multiple problem areas. We developed an online self-report assessment of knowledge and perceived self-efficacy and a telephone-administered standardized patient simulated treatment interview methodology for technique assessment. Study design allowed an independent evaluation of the effects of web-based training, as well as web-based training plus supervision delivered by an experienced cognitive-behavioral supervisor. Our study is also innovative in the development and utilization of standardized patients to measure acquisition of treatment techniques. Standardized patient methodology is widely used in assessing clinical diagnostic techniques in other areas of medicine [35,36], but this approach has been rarely used as an outcome measure in studies of training in mental health treatment methods. Our multi-dimensional assessment design facilitated our collection of systematic data across domains of assessment.

The study included relatively few exclusion criteria. We intentionally sought to optimize external validity and generalizability to the broadest population of mental health providers. Participants consisted of mental health providers from a variety of disciplines, including psychologists, social workers, nurse clinicians, psychiatrists, and others, with varying levels of familiarity with CBT methods.

It is important to recognize that while this training was specifically developed for VHA providers, this approach to providing internet-based training and telephone supervision seems feasible to offer to other groups. Use of volunteer research participants is a potential limitation of the study. However, all trials that consent and randomize individuals run the risk of volunteer bias.

Other design aspects were also aimed to increase generalizability, as recommended by Tunis *et al*. [37] and Glasgow *et al*. [38] for conducting ‘practical clinical trials’ (PCT) in areas of high public health need [3]. In addition to recruiting participants broadly representative of the target training population, the representativeness of organizational settings in which the study is conducted is a component of PCT design. The range of organizational settings in the present study was intentionally broad and included mental health clinics and non-medical center programs such as community-based ‘vet centers.’ A limitation, however, is that not all VHA clinical settings could be included, and it should be noted that many veterans continue to receive health care outside of VHA facilities.

A final PCT principle concerns the use of broad and clinically-relevant outcome measures [37,38]. We attempted to assess a range of outcomes (knowledge, perceived self-efficacy, self-reported implementation, independent rating of technique competence) possible for a study of this type. This was a major focus of our study design. On the other hand, we were required to make choices as to the selection of measures that will inevitably limit our conclusions. Our focus on clinical technique assessment via standardized patient interviews, in particular, was a critical choice for outcomes assessment, the results of which will be evident when the study is unblinded. There are some significant limitations to our assessment procedures. Most of the assessment measures used were study-specific, and have not been validated. We were unable to locate, for example, established measures of provider knowledge that mapped onto the techniques we targeted. We constructed simple self-efficacy ratings linked to elements of the techniques. Because we focused on limited aspects of motivational interviewing, we did not use existing motivational interviewing measurement instrumentation. We also did not include measures of patient outcomes related to the training content (*e.g.*, rates of engagement in the PTSD treatment process as an indicator of the effectiveness of training in motivational interviewing). This was judged too difficult to achieve given the resources of the study, but should be included whenever possible in studies of this type. Overall, in this study, we attempted to balance traditional knowledge-based measures with more potentially valid ‘cutting edge’ measures involving standardized patient interviews and behavioral ratings of clinical techniques [39-41].

## Conclusions

The study described is the first to prospectively evaluate in a controlled, randomized design, the effects of web-based training in core techniques in cognitive-behavioral therapy for veterans with PTSD. The urgent need for practitioners qualified to deliver such treatment highlights the need to develop evidence-based training methods for changing practice, knowledge, and self-efficacy among mental health providers both in large healthcare systems and smaller practices and clinics. Our study will examine the separate but potentially interactive contributions of web-based and non-web based training components, in addition to specific approaches to assessment of knowledge gain, technique development, and perceived self-efficacy changes that occur following each of the training interventions. Few previous studies have employed such a rigorous and well-controlled design for assessing training effects in mental health providers serving those returning from military deployments: an area of extremely high public health need [3].

## Competing interests

The authors declare that they have no competing interests.

## Authors’ contributions

JR and RR co-directed the study, participated in all study aspects, and drafted the manuscript. LM oversaw development of the online training materials, web development, and organized data gathering systems. DG led participant recruitment, collaborated in conduct of assessments, and oversaw gathering of standardized patient simulated interview data. LS participated in web development, study design and outcomes measurement system implementation. AS participated in the design of the study and performed the statistical analysis. All authors contributed to manuscript preparation and read and approved the final manuscript.
